# Long-term results of 157 cemented endoprosthetic reconstructions for tumors of the humerus: a 30-year experience

**DOI:** 10.1016/j.jseint.2026.101700

**Published:** 2026-03-26

**Authors:** Mathangi Sridharan, Christopher Hamad, Danielle Brown, Rishi Trikha, Joseph Kendal, Nicholas M. Bernthal, Lauren Wessel

**Affiliations:** a Department of Orthopaedic Surgery, University of California Los Angeles, Los Angeles, California, USA; b Department of Orthopaedic Surgery, University of California Davis, Sacramento, California, USA; c Department of Orthopaedic Surgery, University of Calgary, Calgary, Alberta, Canada

**Keywords:** Humerus, Endoprosthesis, Long-term outcomes, Limb salvage, Reconstruction, Implant failure

## Abstract

**Background:**

Limb salvage surgery in the upper extremity requires additional focus on soft-tissue repair and reconstruction to preserve functionality, particularly given the different stability needs of upper extremity reconstructions in terms of range of motion and dexterity. Long-term implant survival data following limb salvage surgery of the upper extremity is heterogenous and largely limited to small series. This study assesses the long-term outcomes and causes of failure of endoprosthetic reconstruction for tumors involving the humerus.

**Methods:**

One hundred and fifty-seven consecutive patients who underwent limb salvage surgery via proximal, distal, or total humerus replacement (THR) for musculoskeletal tumors at a single institution between 1980 and 2021 were reviewed. Demographic, oncologic, procedural, and functional outcome data were analyzed with average follow-up of 5.6 years. Implant failure was defined as revision surgery and classified by the Henderson classification; failure excluded expected exchange of modular components. Descriptive statistics and 2-sample *t-tests* were performed on the cohort (Stata, College Station, TX). Patient, implant, and limb salvage survival rates were calculated using implant revision as the endpoint. Kaplan–Meier survivorship analysis was performed on patients based on grade of disease.

**Results:**

A total of 125 proximal humerus, 16 total humerus, and 16 distal humerus endoprostheses were included. The mean age of the cohort at surgery was 43.5 ± 23.9 years. Fifty-eight percent of patients were treated for primary sarcomas. Average follow-up time was 5.7 ± 7.1 years (range: 3 months-31.9 years); 73.3% of the cohort followed up for at least 10 years. There was a 19% rate (30 cases) of implant failure. Failure occurred on average 3.9 ± 4.0 years after surgery. The most common reason for implant failure was tumor progression in 13 cases (8.3%); 5 (3.2%) for soft-tissue failure; 5 (3.2%) for structural failure; 4 for aseptic loosening (2.5%); and 3 for infection (1.9%). 4. Eight percent (8) patients were revised to forequarter amputation, predominantly due to tumor progression. PHRs had an implant-specific survival of 76.0% at 10 years, which exceeded that of distal humerus replacement and THR. THR has lower forward elevation (*P* = .03) and abduction (*P* = .03), to PHR.

**Conclusion:**

The present study confirms the long-term durability of cemented endoprosthetic reconstructions in setting of oncologic reconstruction for massive bone and soft tissue tumors of the humerus. In this series, the most common cause of implant failure requiring revision of the stemmed component in humerus reconstructions is tumor progression, suggesting that oncologic modality of failure is more likely than mechanical failures in this setting.

Given the advancements in early diagnosis, imaging modalities, chemotherapeutic regimens, and surgical techniques, limb salvage is a preferred treatment for sarcomas of the extremities when appropriately indicated.^26^^,^^30^ Limb salvage is frequently achieved with a variety of reconstructive techniques, the outcomes of which are dependent on oncologic as well as mechanical considerations. The Henderson Classification characterizes 5 modes of failure (soft-tissue failure, aseptic loosening, structural failures, infection, and tumor progression) and has been used to evaluate limb salvage reconstruction.^12^^,^^13^ Previous studies evaluating lower extremity cemented endoprosthetic reconstruction demonstrate that the most common mode of failure is mechanical and varies based on anatomical site.^3^^,^^7^^,^^8^^,^^22^^,^^25^ There is limited literature and long-term data on the survivorship of upper extremity reconstructions or their modalities of failure.

While the upper extremity does not have the weightbearing requirements of the lower extremity, the joints in the upper extremity have greater range of motion requirements. Thus, limb salvage surgery of the upper extremity requires additional focus on soft-tissue repair and reconstruction to preserve functionality. The humerus is the most common site of primary bone sarcoma and metastatic bone disease in the upper extremity.^11^ When joint reconstruction is required after resection due to tumor in this location, proximal, distal, or total humerus replacements (PHRs, DHRs, and THRs) are typically utilized with the aim to preserve shoulder, elbow, and hand function. Resection can compromise joint-stabilizing structures, including the rotator cuff, deltoid insertion, joint capsules, and axillary nerve innervation structures; thus, proximal migration of the humeral head or subluxation/dislocation is a common risk after PHR or THR.^5^^,^^15^ Allograft prosthetic composite (APC) and reverse total shoulder arthroplasty (rTSA) have been employed as alternate methods of reconstruction to mitigate these shortcomings, although both methods are not without complication.^9^^,^^15^

In an analysis across 5 institutions, Henderson *et al*^12^ found nonmechanical modes (infection and tumor progression) of failure to be most common in reconstructions involving the humerus, which contrasted with failure modes in the lower extremity. Additional long-term survival and outcomes of endoprosthetic reconstructions of skeletal defects in the humerus can better inform surgeons about pertinent risks and expected results after humerus reconstruction and allow for better patient counseling.

The objective of this study was to examine long-term outcomes of endoprosthetic reconstruction for tumors of the humerus utilizing a large database with up to 30 years of follow-up. Given the differences in weightbearing requirements, we hypothesize that long-term survival after limb salvage surgery for proximal, distal, or total humerus reconstruction for oncologic indications demonstrates high patient and implant survivability, and that unlike lower extremity reconstructions, nonmechanical modes of failure will be more prevalent in this population.

## Methods

All patients that underwent limb salvage surgery with endoprosthetic reconstruction for musculoskeletal tumors of the humerus between December 1980 and December 2021 were retrospectively reviewed. Patients were included if they underwent a PHR, DHR, or THR. Indications for reconstruction were primary or metastatic tumors of the humerus necessitating resection and amenable to limb salvage surgery. Nononcologic indications were excluded. While reverse shoulder arthroplasty reconstructions and APC reconstructions have increased in utilization over the past decade,^9^^,^^15^ these were excluded from this investigation to comment exclusively on long-term outcomes after the historical standard of endoprosthetic reconstructions, which predominantly rely on hemiarthroplasty.

### Data collection

Demographic information collected included date of birth, age, and sex. Oncologic information including primary diagnosis, pre-operative and post-operative chemotherapy and radiation therapy were documented. Tumors were staged at time of diagnosis according to the Enneking system: benign or low-grade, malignant stage I disease; high-grade, stage II disease without metastases; and stage III disease including primary sarcoma that has metastasized, metastatic disease of bone, myeloma, or lymphoma.^6^ All follow-up was performed at the same institution at which the index surgery was performed. Follow-up was recorded until the end of the study period or patient demise.

Outcomes collected include patient survival, incidence of reoperations, margin status, post-operative complications, and shoulder range of motion (forward flexion, abduction, internal rotation (IR), external rotation (ER), elbow flexion–extension arc, pronation, and supination). Post-operative complications included positive margins, superficial or deep infection, instability events (subluxation or dislocation), and local recurrence. Implant failure was defined as any event that required revision surgery, excluded expected change of modular components; implant survival was described relative to this defined event and further classified by Henderson mode of failure.^13^ Time to and mode of failure were analyzed. Institutional review board approval was obtained prior to data collection.

### Surgical technique

Reconstructions were performed by one of 2 board-certified orthopedic oncologists at a single institution. All stems used were cemented. Each PHR was cemented distally in the humeral canal; THR was mated with an elbow joint replacement and cemented into ulnar canal; and the DHR components were cemented proximally in the humeral canal and distally in the ulnar canal. To maintain consistency between groups, all PHRs and THRs were bipolar hemiarthroplasties and reverse total shoulder implants were excluded from this analysis. Perioperative information, including implant modularity and stem length, was obtained from operative reports. The first 34 implants were custom fabricated (between 1981 and 1993). Remaining implants since the early 1990s utilized modular, off-the-shelf titanium systems. Historically, custom and modular prostheses are comparable to each other and thus were analyzed together.^22^^,^^23^ Despite variations in manufacturer and implant design over the entire study period, surgical technique among the 2 operating surgeons remained consistent, specifically regarding tumor resection, cementation, implant management, and soft-tissue reconstruction. Both surgeons perform soft-tissue reconstruction without use of targeted implants; reconstruction is aimed at providing appropriate joint stabilization, specifically at the shoulder girdle and elbow.

### Data analysis

Descriptive statistics were performed on the cohort (Stata, College Station, TX). Functional outcomes between different types of implants were compared with 2-sample *t* tests or Mann–Whitney *U* tests. Patient, implant, and limb salvage survival rates were calculated using implant revision as the endpoint. Kaplan–Meier survivorship analysis was performed on patients based on grade of disease.

## Results

One hundred and fifty-seven patients were included in the final cohort, consisting of 125 proximal humerus (79.6%), 16 total humerus (10.2%), and 16 distal humerus endoprosthetic reconstructions (10.2%). Thirty-six patients (22.9%) were <18 years of age on presentation. Eleven patients had expandable endoprostheses. The mean age at surgery was 43.5 ± 23.9 years, with 65 (41.4%) females and 92 (58.5%) males.

The tumors were low grade (IA/IB) or benign in 24 (15.3%) patients, high-grade (IIA/IIB) in 63 (40.1%), and metastatic or stage III in 71 (44.6%) (Table I). Ninety-one patients (57.9%) had primary sarcomas (Table II). Average follow-up time was 5.7 ± 7.1 years (range: 3 months-31.9 years). Thirty-four (21.6%) implants were custom, and 123 (78.3%) implants were modular. Seventy-seven patients (49.0%) underwent pre-operative chemotherapy, and 26 (16.5%) received pre-operative radiation therapy to the surgical site. Post-operatively, 87 (55.4%) patients received chemotherapy or radiation therapy. Impacts of adjuvant therapies on outcomes was difficult to analyze due to missing data on regimens in retrospective chart review and when treated by outside medical and radiation oncology teams.

**Table I:** Survival data following upper extremity endoprosthetic reconstruction.

Survival group	5 yr	10 yr	15 yr	20 yr	25 yr	30 yr
Implant survival						
Custom (N = 34)	67.2%	47.2%	23.6%	23.6%	23.6%	23.6%
Modular (N = 123)	85.8%	83.2%	78.3%	78.3%	78.3%	-
Total humerus (N = 16)	83.3%	33.3%	33.3%	33.3%	33.3%	33.3%
Distal humerus (N = 16)	74.9%	74.9%	37.4%	37.4%	37.4%	-
Proximal humerus (N = 125)	81.5%	76.0%	66.2%	66.2%	66.2%	66.2%
Overall (N = 157)	81.1%	71.6%	59.9%	59.9%	59.9%	59.9%
Patient survival						
Low grade or benign (N = 23)	93.7%	86.5%	74.2%	74.2%	74.2%	74.2%
High grade IIA/IIB (N = 63)	62.5%	49.7%	37.6%	33.5%	33.5%	33.5%
Stage III/metastatic (N = 71)	25.2%	12.6%	3.2%	3.2%	-	-

**Table II:** Distribution of tumor diagnoses by group.

Group 1 Benign, grade IA/B (N = 23)	Group 2 Grade IIA/B (N = 63)	Group 3 Grade III, metastatic (N = 71)
Chondrosarcoma^12^	Osteosarcoma (35)	Metastatic disease (51)
Giant cell tumor^11^	Chondrosarcoma^13^	Osteosarcoma^13^
	Ewing's sarcoma^10^	Myeloma^2^
	Malignant fibrous histiocytoma^3^	Malignant schwannoma^1^
	Rhabdomyosarcoma^2^	Intraosseous leiomyosarcoma^1^
		Malignant fibrous histiocytoma^1^
		Lymphoma^1^
		Fibrosarcoma^1^

### Patient survival

**Figure 1:**
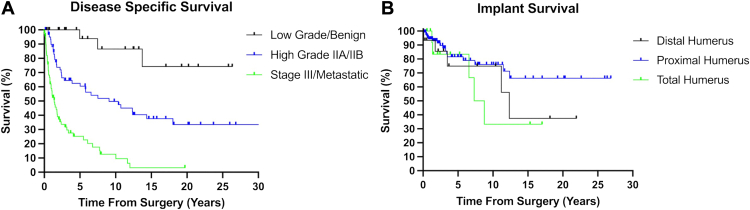
Kaplan–Meier survival curves demonstrating disease-specific patient survival (**A**) and implant survival using revision of the stemmed components as the endpoint (**B**).

Kaplan–Meier survivorship analysis revealed 93.7% disease-specific survival at 5 years for patients with low-grade or benign disease, with only one patient dying of disease due to pulmonary complications of giant cell tumor; survival dropped to 86.5% at 10 years (Table I, Fig. 1*A*). For patients with high-grade IIA/IIB disease at time of surgery, survival was 62.5% at 5 years and fell to 37.6% by 15 years. Disease-specific survival was lowest at all time points for patients with stage III primary sarcoma or metastatic disease, with survival of 25.2% at 5 years, 12.6% at 10 years, and 3.2% by 15 years.

### Implant failure

**Figure 2:**
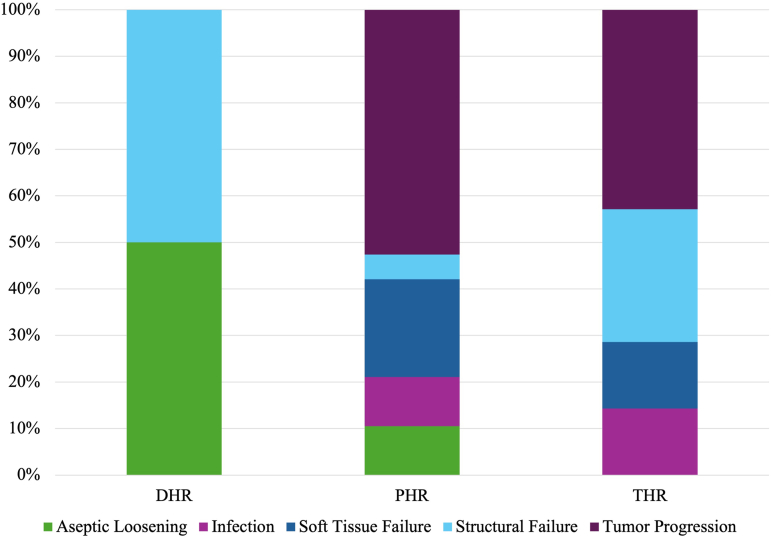
Kaplan–Meier survival curve demonstrating implant survival after limb salvage surgery. *DHR*, distal humerus replacement; *PHR*, proximal humerus replacement; *THR*, total humerus replacement.

Overall, there was a 19.1% rate (30 cases) of implant failure, defined as requiring revision surgery. Failure occurred on average 3.9 ± 4.0 years after surgery. The most common reason for implant failure was tumor progression in 13 cases (8.3%); 5 (3.2%) for soft-tissue failure; 5 (3.2%) for structural failure; 4 for aseptic loosening (2.5%); and 3 for infection (1.9%), caused either by *C. acnes* and *H. influenzae* (Fig. 2).

Twelve patients (7.6%) experienced a subluxation or dislocation event, of which 7 were managed with closed reduction or observation; this is an expected complication of PHR. The remaining 5 required revision soft-tissue reconstruction. There were 3 (1.9%) cases of superficial wound infection treated with antibiotics and 1 (0.6%) periprosthetic olecranon fracture treated nonoperatively, At final follow-up, 8 (5.1%) upper extremity endoprosthesis implants were revised to forequarter amputation, of which 7 (4.4%) were performed for tumor progression and 1 (0.6%) was for structural failure followed by tumor progression. Of note, 4 (2.5%) patients in the total cohort had positive margins after tumor resection, of which 2 had tumor progression requiring amputation. Six patients that experienced implant failure (4 THR, 2 PHR) were converted to rTSA. The modes of failure that lead to conversion were 1 for infection, 4 for structural failure, and 2 for instability secondary to aseptic loosening with concurrent joint pain. The average number of reoperations in the entire cohort was 0.25 (range: 0-3).

### Implant survival

**Figure 3:**
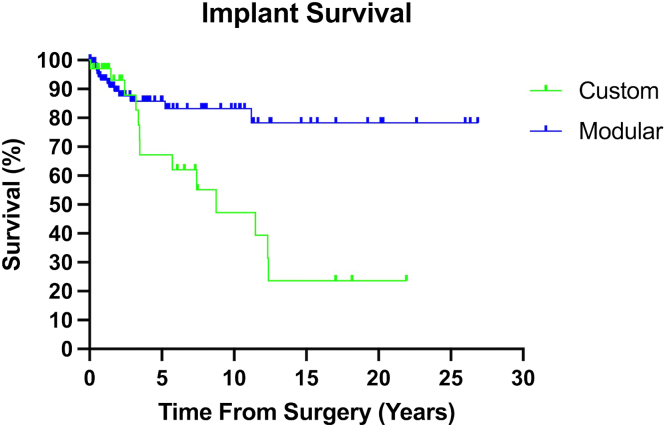
Henderson mode of failure by implant type.

Overall implant survival at 10 years was 71.6% and 59.9% by 30 years (Table I). However, there was a clear discrepancy of longevity based on anatomic site. PHRs demonstrated the highest survival with 81.5% survival, which dropped to 66.2% by the end of the study period (Fig. 1*B*). THRs had 83.3% survival at 5 years, which fell steeply to 33.3% by 10 years. The initial high survival was likely driven by a limited sample size. DHRs behaved similarly with 74.9% survival at 5 years, which fell to 37.4% by 15 years. Modular implants demonstrated better survival than custom-designed components at most time points in the study (Fig. 3); the difference deviated largely at 10 year follow-up, with custom implants at 47.2% and modular implants for 83.2% survival.

### Functional outcomes

For patients that underwent PHR, average shoulder range of motion was limited with forward elevation of 38.7° ± 39.9°, abduction of 37.8° ± 35.3°, IR of 81.7° ± 20.9°, and ER of 16.9° ± 26.3°. For those that underwent THR, average shoulder range of motion was limited with forward elevation of 31.4° ± 34.9°, abduction of 32.8° ± 29.8°, IR of 64.2° ± 34.5°, and ER of 40.8° ± 44.1; elbow flexion–extension arc was 84.2° ± 38.7°, pronation 89.2° ± 2.8°, and supination 81.2° ± 24.9°. For patients that underwent DHR, elbow flexion–extension arc was 70.6° ± 30.5°, pronation 82.5° ± 19.9°, and supination 88.9° ± 2.9°.

Compared to PHR, THR had lower forward elevation (*P* = .03) and abduction (*P* = .03), but similar IR (*P* = .3) and similar ER (*P* = .2). Compared to DHR, THR had similar elbow flexion (*P* = .29), elbow extension (*P* = .65), and supination (*P* = .33), but significantly higher pronation (*P* = .09).

## Discussion

The current study utilizes a large retrospective cohort with long-term follow-up to demonstrate the longevity of cemented endoprosthetic reconstructions in cases of massive bone and soft tissue loss in the humerus. Previous examinations have demonstrated that proximal humerus endoprostheses have high durability with a relatively low complication profile, though these have often been limited by low sample sizes and limited follow-up.^4^ This study offers one of the largest single institutional cohorts of upper extremity endoprosthesis with up to 30 years of survival data. Overall, our findings suggest that PHR is a durable, effective limb-salvage option for appropriate patients with upper extremity tumors of the humerus. THR and DHR were also demonstrated to be effective options for limb-sparing surgery for indicated patients, though their durability is less than that of PHR.

These findings are especially true for patients with high-grade or metastatic disease, for whom implant survival exceeds patient survival. Tumor progression was the most common indication for revision, and, as expected, patient survival differed according to presenting diagnosis, with low-grade and benign lesions consistently demonstrating higher survival rates than high-grade or metastatic disease. This demonstrates that reconstructions in appropriate patients may be durable enough to last for their remaining lifespan and minimize the need for revision surgery, which may be particularly pertinent in patients with metastatic bone disease who are now outliving traditional fixation techniques.^17^ PHR implants had survival comparable, but not exceeding, that of patients with low-grade or benign disease. Despite the durability of PHR, this indicates that such patients should be counseled at regarding the need for modular component exchange in their lifespan.

Although long-term implant survival of upper extremity endoprosthesis is greater than that of lower extremity endoprosthesis, our study also demonstrated clear discrepancy of upper extremity implant durability based on anatomic site. Specifically, our results demonstrated that PHR has greater long-term survival than DHR and THR, which is consistent with prior literature.^18^^,^^19^^,^^25^ Historically, THR and DHR are difficult to study given the rarity of tumors necessitating these reconstructions; this is reflected in our study, with PHR being by far the most prevalent humerus endoprosthesis. Previous analyses of total humerus endoprosthesis implanted for primary upper extremity bone disease have demonstrated survival rates that are significantly lower than that of PHR^28^; survival predictably decreases with extended follow-up.^2^^,^^21^ The difference in survival based on the anatomic site within the humerus may be due to the extensive bone resection and lack of soft tissue attachments in THR. Fixation of the total humerus implant is reliant on the cemented stem in the ulnar canal and the prosthetic ulnohumeral joint articulation, which is more rotationally unstable and has a longer lever arm than endoprosthetic implants that have cemented fixation within the humeral canal.

DHR similarly has lower survival rates, with the most common cause of failure being aseptic loosening.^14^ This is likely due to the increased shear and rotational forces commonly seen at the elbow. Previous literature defining failures in endoprosthetic reconstruction confirms this trend, despite the rarity of tumors in this anatomic location.^12^ Henrichs et al^14^ reviewed 12 distal humerus endoprosthetic reconstructions and found implant survival to be 82% and 64% and 2 and 5 years after surgery. Implant survival in the THR and DHR groups may be improved with allograft soft-tissue reconstruction to confer greater stability, improved modular designs, or consideration of custom implants based on the tumor being resected. Biomechanical studies evaluating implant stability based on location in the humerus may offer insight on how to improve survival for THR and DHR. For PHR specifically, the increasing use of rTSA for proximal humerus reconstruction offers an alternative that may confer improved survivability and functionality; however, research with long-term follow-up on rTSA PHR is still needed.

Despite a high rate of limb preservation and implant durability, constructs were revised at a rate of 19%, most commonly due to nonmechanical modes of failure, namely tumor progression. This differs from lower extremity endoprosthesis, in which mechanical failure is most common.^7^^,^^25^ Previous literature has similarly shown that mode of failure after endoprosthetic reconstruction differs based on anatomic site, in both upper and lower extremity. Henderson et al^12^ performed a multi-institutional examination of endoprosthetic reconstructions and found similar results for PHRs, though infection and soft-tissue failure modes of failure were most prevalent. While mechanical failure was most common for THR and DHR, these procedures have been historically underpowered due to the rarity of tumors necessitating such reconstructions. Given the anatomic considerations for these reconstructions described earlier, it is likely that THR and DHR are more prone to aseptic loosening compared to PHR.^14^

The complication profile in this cohort on par with what has been previously reported in upper extremity literature, although existing studies are limited by follow-up and sample size. Schneider et al^21^ previously demonstrated 93% limb survivorship at 1- and 5-year follow-up after total humerus endoprosthetic reconstruction across 19 years at a single institution. Cannon et al^4^ previously reviewed 83 proximal humerus endoprosthetic reconstructions over a 15-year period and demonstrated a relatively low complication profile, with no cases of aseptic loosening; 2 patients with deep infections; and nearly 25% with proximal migration of the prostheses. Our study did demonstrate that 8% of the cohort had a dislocation or subluxation event. Instability events are not uncommon after upper extremity endoprosthesis and are often increased in resections that compromise the rotator cuff and other soft-tissue stabilizers.^9^^,^^19^ In addition, our rate of upper extremity endoprostheses progression to revision amputation is consistent with prior literature and is not surprising, given that tumor progression was the most common cause of failure of endoprosthesis in this study.^24^ These outcomes demonstrate long-term follow-up is required for endoprosthetic reconstruction of the upper extremity to adequately identify and treat failures.

Our results also demonstrated that modular implants have improved survival in comparison to custom implants over the study period. Custom implants are often required for very complex, tumor cases requiring extensive bone and soft-tissue resection, which may bias them toward poorer outcomes. In addition, custom implants historically demonstrated poorer outcomes because of inflexibility in intraoperative decision-making and minimal room for intraoperative adjustments. This is mirrored in our data, as the use of custom implants was predominantly concentrated in the early years of this study, with the transition to modular implant usage mirroring the development of modular systems for upper extremity endoprosthesis. Although implant design has evolved over the study period to allow greater use of modular systems even in complex surgical situations, the use of custom implants is still indicated based on the patient in oncologic settings.^22^^,^^27^ The longevity of contemporary modular implants has been demonstrated in previous literature for lower extremity endoprosthesis, including distal femur and proximal tibia, over a study period that is directly comparable to this study.^22^^,^^29^ Our data suggests that this extends to the upper extremity as well. The advantage of modular implants lies in the ability to tailor components to the extent of reconstruction intraoperatively.

The current study demonstrated similar, limited shoulder range of motion after hemiarthroplasty reconstruction among PHR and THR cohorts, with average shoulder elevation and abduction.^1^^,^^4^ This finding is likely secondary to both a lower sample size in the THR cohort as well as the exclusion of reverse total shoulder reconstructions in both groups to maintain consistency between cohorts. Existing literature demonstrates that despite the durability of hemiarthroplasty endoprosthetic reconstruction, shoulder range of motion remains notably limited post-operatively, likely due to extensive resection of soft-tissue restraints that typically contribute to glenohumeral stability.^4^^,^^9^ Furthermore, elbow range of motion did not differ significantly between THR and DHR in this current study. Although flexion is relatively well preserved, extensor lag is common after total or distal humerus reconstruction.^14^^,^^21^ Again, this is likely due to circumferential soft-tissue resection compromising the extensor mechanism of the elbow. In addition to counseling patients accordingly, future areas of study should include effective extensor mechanism reconstruction and early post-operative rehabilitation to preserve motion. While a significant difference in average post-operative forearm pronation was seen between the 2 cohorts, THR and DHR are relatively underpowered in this study; as such, this finding requires further validation and is not necessarily clinically significant. A growing utilization of rTSA as well as APC reconstructions for extensive proximal humerus bone loss has preliminarily demonstrated improved range of motion and functional outcomes.^9^^,^^10^^,^^15^^,^^16^^,^^20^ These techniques fell outside the scope of this study.

The current study has several limitations in addition to the inherent limitations of a retrospective study. This study only included procedures performed by 2 surgeons at a tertiary referral center, given the expertise needed to provide complex oncologic care. While this standardized intraoperative and post-operative protocols, this also limited the generalizability of results. For example, exclusively cemented stem fixation was utilized in all humerus reconstruction in this study; thus, the results are only applicable to this specific mode of fixation. Given the relative rarity of upper extremity tumors requiring endoprosthetic reconstruction, sample sizes are limited for THRs and DHRs. Given the importance of long-term data for survival, this study also includes excluded rTSAs, as these require larger long-term datasets from which to draw conclusions. Further prospective studies on the use of rTSA in the setting of extensive proximal humerus bone loss and functional outcomes compared to endoprosthetic hemiarthroplasty are undoubtedly needed. Furthermore, advances in therapies and surgical technique over the course of our study period may impact the generalizability of our results. This institution will publish a larger cohort of rTSA endoprosthetic reconstructions at a future date.

## Conclusion

The current study represents one of the largest data captures of upper extremity reconstructions specific to the humerus, with long follow-up data. It offers insights on implant survival and limb salvage for historically understudied humerus endoprosthesis over a 30-year experience, with tumor progression being the most common cause of failure. This demonstrates the long-term durability of cemented endoprosthetic reconstructions for musculoskeletal tumors of the upper extremity and provides some validation that they remain a durable reconstructive option.
